# A Heterogeneous RISC-V Processor for Efficient DNN Application in Smart Sensing System

**DOI:** 10.3390/s21196491

**Published:** 2021-09-28

**Authors:** Haifeng Zhang, Xiaoti Wu, Yuyu Du, Hongqing Guo, Chuxi Li, Yidong Yuan, Meng Zhang, Shengbing Zhang

**Affiliations:** 1 National & Local Joint Engineering Research Center for Reliability Technology of Energy Internet Intelligent Terminal Core Chip, Beijing Smart-Chip Microelectronics Technology Co., Ltd., Beijing 100192, China; zhanghaifeng@sgitg.sgcc.com.cn (H.Z.); yuanyidong@sgitg.sgcc.com.cn (Y.Y.); 2 School of Cybersecurity, Northwestern Polytechnical University, Xi’an 710072, China; xiw26@mail.nwpu.edu.cn; 3 Engineering and Research Center of Embedded Systems Integration (Ministry of Education), Xi’an 710129, China; 2019262266@mail.nwpu.edu.cn (Y.D.); guohongqing@mail.nwpu.edu.cn (H.G.); lichuxi@mail.nwpu.edu.cn (C.L.); zhangsb@nwpu.edu.cn (S.Z.); 4 National Engineering Laboratory for Integrated Aero-Space-Ground-Ocean Big Data Application Technology, Xi’an 710129, China; 5 School of Computer Science, Northwestern Polytechnical University, Xi’an 710129, China; 6 School of Software, Northwestern Polytechnical University, Xi’an 710129, China

**Keywords:** sensing system, dnn, intelligent computing architecture, RISC-V, VLIW, SIMD

## Abstract

Extracting features from sensing data on edge devices is a challenging application for which deep neural networks (DNN) have shown promising results. Unfortunately, the general micro-controller-class processors which are widely used in sensing system fail to achieve real-time inference. Accelerating the compute-intensive DNN inference is, therefore, of utmost importance. As the physical limitation of sensing devices, the design of processor needs to meet the balanced performance metrics, including low power consumption, low latency, and flexible configuration. In this paper, we proposed a lightweight pipeline integrated deep learning architecture, which is compatible with open-source RISC-V instructions. The dataflow of DNN is organized by the very long instruction word (VLIW) pipeline. It combines with the proposed special intelligent enhanced instructions and the single instruction multiple data (SIMD) parallel processing unit. Experimental results show that total power consumption is about 411 mw and the power efficiency is about 320.7 GOPS/W.

## 1. Introduction

Traditional Internet of Things (IoT) devices are usually responsible for data measurement, data collection, and pre-processing tasks. Due to the limitation of bandwidth, the huge amount of data generated by the edge devices cannot be transmitted to the cloud for further AI intelligent computing. Extracting features from sensing data by DNN in the sensing system is challenging as deploying intelligent applications requires the trade-off between real-time and high efficiency in the resource-limited edge devices. At this stage, the widely-used micro-controller-class processors in sensing system, such as MCS51 and STM32, accomplish given tasks without an operating system and with limited memory and low processing capacity. Because of the poor performance of the micro control unit (MCU), deploying neural networks directly on micro-controller-class processors faces many difficulties. Notably, intelligent applications impose strict requirements on: (1) high computing performance, (2) low power consumption, and (3) flexible configuration [1]. Therefore, it is necessary to design advanced processors equipped to the sensing system to satisfy the demands of deploying DNN with balanced performance metrics. Therefore, it is necessary to design advanced processors equipped with the sensing system to satisfy the demands of deploying DNN with balanced performance metrics.

In the edge applications of feature recognition and data abstraction, DNN calculation has to be executed with sub-millisecond latency, which requires the computing performance of the sensors with large data sampling interval is not less than 3 GOPS. Presently, typical neural network hardware accelerator is designed for servers or high-performance computing centers [2,3,4,5,6,7], which focus on accelerating performance and ignore resource consumption and power budget.

Although they have high computing performance, they cannot be used in the resource and power constrained sensing system.

To alleviate the poor-performance problems, a number of studies have been undertaken to accelerate DNN implementations by designing hardware-accelerated intelligent computing architecture for sensing system.

Some researches exploit the property of DNN to reduce latency by using the parallel characteristics of special acceleration circuit design, such as [8,9,10,11,12,13,14]. Yet these works ignore that the whole power consumption exceeds budget. At present, most of the ideal edge devices in the sensing system are powered by battery, which requires power consumption around hundreds of milliwatts. The power budget consists of average power consumption and instantaneous power consumption. Limited instantaneous power consumption will lead to a decrease in computing performance.

The other part of the researches are devoted to use optimization method to improve energy efficiency ratio. For example, the throughput of the architecture proposed in [15] reaches 59.52 GOPS at 120 MHZ, and the power consumption is 1.35 W. The same phenomenon appears in [16,17,18]. Although these works on intelligent computing architecture for edge applications meet the requirements of real-time performance, the balance between energy efficiency ratio and overall power consumption cannot be achieved. In short, these previous works focus on the energy efficiency of the embedded intelligent computing. For the limited power budget in specified AIoT applications, the lack of system level energy and power optimization are still needed to be researched.

The network deployed in the sensing system of the feature extraction is not a single structure. Different network characteristics require corresponding neural network structures for better support. The computing architectures designed in [19,20] rely on single network structure or are only for a single application field, resulting in poor adaptability. So these works are difficult to apply to other application scenarios or different network structures. For example, it will cause a lot of waste of resources when the neural network structure with a large number of channels is used to accelerate the calculation of small-channel networks. In the intelligent application of feature extraction of sensor data, the obtained data have various characteristics from the perspective of space, so the network structures are adapted to different scenarios, which puts forward requirements for configuration flexibility. In terms of time, as the amount of sensor data increases, the analysis strategy needs to be adjusted and the acquisition algorithm needs to be modified, resulting in higher demands on flexibility. Most of them are dedicated circuits designed for a specific network, and their flexibility is difficult to support the analysis of multi-source data.

It is, therefore, extremely challenging to deploy DNN calculation flexibly on sensing system with large power consumption constraints and real-time requirements, which is to adapt to the application requirements of feature extraction and further realize the efficient deployment of DNN model on edge devices.

## 2. Motivation

As the large sampling interval of the edge devices in the sensing system, the limitation of low power consumption is the main areas of concern, and the requirement for improving the calculation performance is relatively low. Most of the common embedded processors are simple MCU, which usually use Harvard structure. In accelerator mode, a lot of redundant data transmissions are inescapable. Previous work has found that memory access energy consumption accounts for the main part (76.3%) of the energy consumption of DNN accelerators [21,22]. The power consumption of transmitting 32-bit data from memory is three orders higher than that of addition operations [23]. In addition, the utilization of the computing unit is limited by the high latency and low bandwidth of memory access [24], since the computing unit remain idle, waiting for data to be transferred from memory.

**Figure 1:**
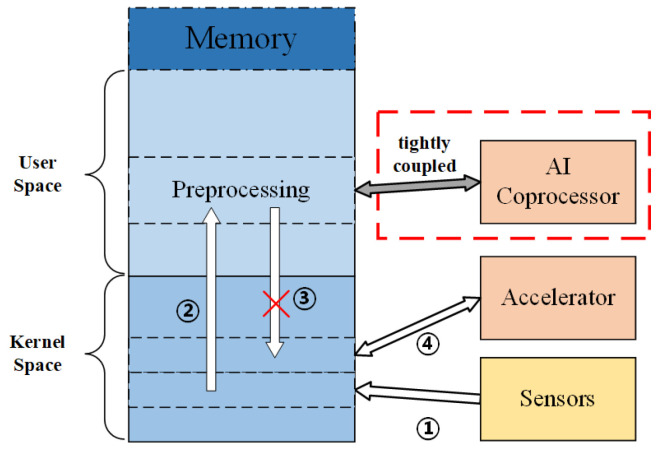
Data movement in the whole system.

As is shown in Figure 1, it is a complex round trip for data movement since the storage of loosing coupling architecture is independent. Firstly, the sensor data are transmitted to the kernel space of memory through the bus, and then transferred to the user space for preprocessing. After the preprocessing has been completed, it is necessary to move the data from the user space to the kernel space and then to drive the accelerator under the system call mode. The accelerator with private storage fetch the data from the kernel space for intelligent computing. The accelerator with private storage fetches the data from the kernel space for intelligent computing. Massive redundant data movements are incurred, such as the third step in Figure 1, which causes significant energy consumption. The design in this work is a tightly coupled architecture, thus the coprocessor and the CPU share the key resources, such as register files, memory, and cache. The intelligent components work in the form of coprocessor and fetch data directly from the user space, thereby improving the efficiency of data access and reducing energy consumption.

The neural network is in a period of rapid development, and the network structure is constantly updated. The key problems in designing a tightly coupled coprocessor for intelligent computing are data storage access and dataflow maintenance. The core of data storage access is that convolution calculation is high dimensional, but the off-chip storage accesses are through one-dimensional linear address, which leads to the dispersion of data access. In addition, dataflow needs to be reorganized on the chip, which puts forward further requirements for the flexibility of data scheduling [22]. In order to support the mapping of different dataflows and various network structures, it is necessary to calculate the parallel features according to the network structure information and the scheduling relationship, and then determine the data path and data access features. To satisfy the reconfigurable requirements, most of the existing intelligent computing structures rely on hardware dynamic scheduling. By modifying the register to adjust to the data path, a large number of hardware scheduling logics have been added to the computing structure, which introduces a very large hardware complexity and energy consumption. Although many optimization methods are used in Eyeriss, the cost of control of the data path is still high [2]. In edge applications with limited resources and power consumption, the scheduling space is limited, thus the flexibility is insufficient.

The complexity of the dataflow maintenance by hardware calculation is high, whereas the software is more suitable for scheduling. From the point of view of the instruction, the pipeline is the execution process of overlapping instructions. Relying on the software technology for static discovery parallel during compilation, the relationship between each calculation and the previous calculation is controlled by the compiler, which controls how the data are moved. The method reduces so much power consumption that higher energy efficiency can be obtained. The irrelevant operations in the pipeline are analyzed and encapsulated into VLIW to complete the static scheduling. For example, in the process of DNN calculation, the next data-block can be transmitted while the convolution calculation of the current data-block is carried out. This irrelevant relationship is so fixed that it is easy to maintain by VLIW. At the same time, SIMD structure is used for the pipeline level with a large number of data-level parallelism so as to achieve the balance between algorithm adaptability and computational performance.

This paper proposes a dedicated intelligent enhanced RISC-V instruction subset which supports adaptive structure mapping and dataflow maintenance, thereby reducing hardware complexity. Under the tightly coupled architecture, the VLIW + SIMD structure is used to further realize the instruction-level parallelism and the data-level parallelism, which meets the balanced performance metrics including low power consumption, low latency, and flexible configuration. The contributions of our work are summarized as follows:

1. We design a mapping method to realize the deployment of different dataflows, thereby satisfying the requirements for configuration flexibility;
2. We propose a lightweight tightly coupled architecture, VLIW ISA based SIMD architecture (VISA), achieving a balanced performance metrics including low power consumption, low latency and flexible configuration;
3. We develop a RISC-V dedicated intelligent enhanced VLIW instruction subset to maintain various dataflows and support different DNN calculations.

## 3. Mapping Method of Data Flow

Common DNN models have some typical characteristics, including computational characteristics and dataflow characteristics. In this section, we analyze the typical features in details and optimize some operations to guide the design of the VLIW and tightly coupled processor.

### 3.1. Analysis of Calculation Consistency

The DNN model is usually composed of some basic calculation layers. The basic calculations mainly include convolution, activation function, batch normalization (BN), pooling, and Softmax. Except for Softmax, the rest of the calculations use hardware modules for calculation. Because Softmax often uses exponents, divisions, and other algorithms which are relatively complex to implement in hardware, and are often at the last layer in the classification network, the Softmax calculation is implemented by the software.

#### 3.1.1. Convolution

The convolution operation is to slide the convolution kernel on the input feature map, which multiplies the corresponding data in the current window, and then add them up. Assuming that the window size is $K_{c}\;*\;K_{r}$, if there are $N_{in}$ input channels, a pixel of the output feature map is obtained by adding the multiplication results of $N_{in}\;*\;K_{c}\;*\;K_{r}$. The convolution operation can be expressed as Formula (1):

(1) $$Output_{\left(r,c,n\right)}=\sum_{m=0}^{Nin}\sum_{i=0}^{Kc}\sum_{j=0}^{Kr}{Input_{\left(x,y,m\right)}}^{\prime }*Weight_{\left(r,c,x,y,m,n\right)}+\;bias_{\left(n\right)}$$

Note: *m*, *n* denote the input and the output channel respectively. *i*, *j* denote the size of convolution kernel. *r* and *c* denote the size of output. *x* and *y* represent the size of output feature map.

Convolution can be carried out by utilizing the parallelism of input channels. The data of multiple input channels can be organized into vectors, and the corresponding weights can also be organized into vectors for calculation. The input data among multiple output channels are the same, but the index of weights is different.

#### 3.1.2. Batch Normalization

The normalization methods mainly include: local response normalization (LRN), local comparison normalization (LCN) and BN. In recent years, BN has gradually replaced LRN and LCN. BN calculation can be summarized as the Formulas (2)–(4):

(2) $$Output_{\left(i,j,k\right)}=\frac{Input_{\left(i,j,k\right)}-mean_{\left(k\right)}}{\sqrt{var_{\left(k\right)}+0.00001}}*\alpha_{\left(k\right)}+\beta_{\left(k\right)}$$

(3) $$mean_{\left(k\right)}=\frac{1}{NHW}\sum_{n=1}^{N}\sum_{h=1}^{H}\sum_{w=1}^{W}x_{\left(k,n,h,w\right)}$$

(4) $$var_{\left(k\right)}=\sqrt{\frac{1}{NHW}\sum_{n=1}^{N}\sum_{h=1}^{H}\sum_{w=1}^{W}{\left(x_{\left(k,n,h,w\right)}-mean_{\left(k\right)}\right)}^{2}+\epsilon }$$

Note: $Input_{\left(i,j,k\right)}$ or $Output_{\left(i,j,k\right)}$ represents the pixel (*i*, *j*) of the *k*th input/output channel in the output feature map. *H*, *W* represent the number of rows and columns of the feature map. *N* represents the number of training samples. bn_weight and bn_bias are obtained after training.

In order to avoid a large number of division operations, we can treat $\frac{1}{\sqrt{var_{(k)\;+\;0.00001}}}$ as a multiplier, which is calculate by CPU. BN calculation can be re-summarized as the following Formulas (5) and (6):

(5) $${Output_{\left(i,j,k\right)}}^{\prime }=\left(Input_{\left(i,j,k\right)}-mean_{\left(k\right)}\right)*\gamma_{\left(k\right)}+\beta_{\left(k\right)}$$

(6) $$\gamma_{\left(k\right)}=\frac{1}{\sqrt{var_{(k)\;+\;0.00001}}}*\alpha_{\left(k\right)}$$

Since mean, var, bn-weight, bn-bias, and the convolution calculation bias are shared with all pixels on a output feature map, $mean_{\left(k\right)}$, and $bias_{\left(k\right)}$ are fixed parameters in a output channel. Therefore, the calculation formula is as Formulas (7) and (8):

(7) $${Output_{\left(i,j,k\right)}}^{\prime \prime }=\left(CONV_{\left(i,j,k\right)}-{bias_{\left(k\right)}}^{\prime \prime }\right)*\gamma_{\left(k\right)}+\beta_{\left(k\right)}$$

(8) $${bias_{\left(k\right)}}^{\prime }=bias_{\left(k\right)}-mean_{\left(k\right)}$$

Note: $Input_{\left(i,j,k\right)}$ is the final multiply-accumulate (MAC) result of convolution calculation.

After the MAC of convolution calculation is completed, the new bias is added to obtain a new convolution result. The BN calculation is finally summarized as Formula (9):

(9) $$Output_{\left(i,j,k\right)}={Input_{\left(i,j,k\right)}}^{\prime }*\gamma_{\left(k\right)}+\beta_{\left(k\right)}$$

Note: ${Input_{\left(i,j,k\right)}}^{\prime }$ is the convolution calculation result after bias adjustment. $\gamma_{\left(k\right)}$,$\beta_{\left(k\right)}$ is new parameters for the BN layer.

In summary, through the pretreatment of parameters and the optimization of calculation, the original complex BN is transformed into a similar operation as convolution. The combination of calculation parameters cannot only reduce the amount of calculations, but also reduce the errors caused by parameter fixed point representation, and improve the accuracy of the calculation. In addition, BN calculation can be carried out by convolution calculation unit to simplify the structure.

### 3.2. Data Flow Scheduling Analysis

As is well known, the convolution can be expressed as a six-fold nested-loop, which contains numerous MAC computations. Due to the limited on-chip storage, a large number of parameters for the convolution cannot be stored on the chip at the same time. Different loop-unrolling ways construct a variety of dataflow patterns, which makes the degree of the data reuse varies greatly. Flexible dataflow scheduling is an effective methods to improve the computational performance of DNN as it can achieve better data reuse in the condition of limited bandwidth. The dataflow scheduling of convolution calculation in this paper is based on the analysis of the three patterns of DI∖DO∖DK [25].

**Figure 2:**
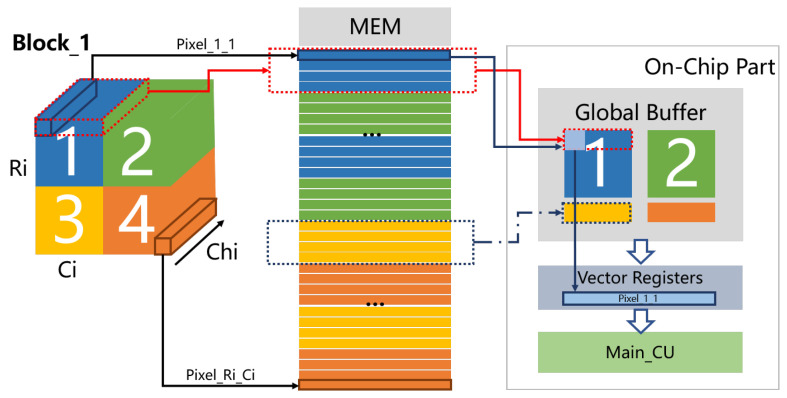
The placement and delivery forms of feature map data.

The data index contains three dimensions: block, row, and column. The data are placed in the off-chip memory by blocks, each block contains T channels. The organization in each block is shown in Figure 2, Ri, Ci, and chi represent the number of rows, columns, and channels of the input feature map, respectively. The data of eight input channels in each pixel are expressed as one row of memory. In memory, the eight input channels of each pixel are continuous and stored from column to row. Each transmission bursts at least one pixel of the data.

Three types of the dataflow correspond to three types of loop unrolling modes. The internal minimum cycle is fixed, and the window is the minimum unit. Therefore, all the data in sub-block1 are transmitted to the chip first, so as to transmit the first calculated demand data as soon as possible to establish the pipeline. The global buffer is set in the form of a row buffer to store sub-block1, sub-block2 and the next row data, that is, a total of K + 1 rows. Each calculation of the main computing unit will use a pixel data. Adjusting the data index address according to the calculation requirements.

**Figure 3:**
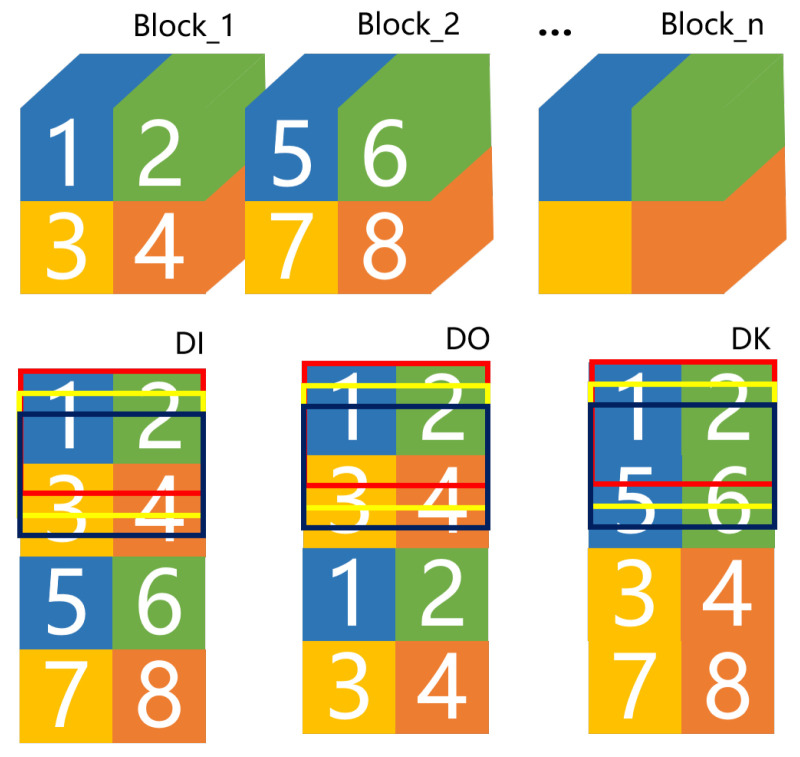
Three dataflow scheduling patterns.

For the three dataflow patterns of DI∖DO∖DK, the difference of input data scheduling is shown in Figure 3. The rectangular boxes represent the filling of data in the global buffer. The movement of each rectangular box indicates the change of global buffer data filling. In DI∖DO patterns, the data is scheduled based on units of the entire feature map. However, DI traverses all input channels firstly. Each block (e.g., Block_1) is transferred from the memory outside the chip in sequence until the first set of output channels is computed. DO traverses all output channels firstly, so the partial sum of all output feature maps is computed and superimposed. DO preferentially traverses all output channels, and block1 is transferred from the memory outside the chip to calculate the partial sum of a set of output channels. Since global buffer cannot store the whole graph data, it is necessary to transfer the data of block1 again to compute the partial sum of the next set of output channels, and to accumulate the partial sum to form output feature maps. Obviously, in DK pattern, the data are scheduled based on the row instead of the entire feature map. After calculating the first K rows of data, it switches to the next block to traverse the input channels. As if the size of feature map is too large, which the whole row cannot be stored in the buffer, each row will be divided into sub-rows.

## 4. VISA: A Lightweight Pipeline Integrated Deep Learning Architecture

The lightweight pipeline integrated architecture proposed in our work mainly includes the dedicated intelligent enhanced VLIW extended instruction set architecture and the SIMD computing architecture.

### 4.1. Architecture Overview

**Figure 4:**
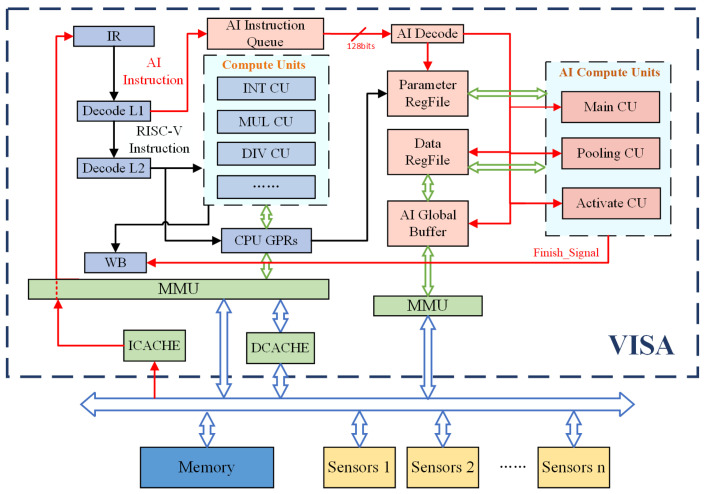
Overall architecture.

The system architecture of this design is shown in Figure 4. The intelligent acceleration components are integrated into the main pipeline in the form of coprocessor. Sensors are mounted on the same bus as processors and memory. The main processor pipeline and the intelligent calculation coprocessor, respectively, access the memory through their respective MMUs. Main processor and intelligent coprocessor share data through the shared area in memory. As shown in Figure 1, the data from the sensor will be first transmitted to the kernel space of memory through the bus, and then moved to the shared area of the user space. The preprocessing program is run by the main processor, and then the intelligent acceleration operation is carried out by the intelligent coprocessor. The final result data are put back to the memory, waiting for the next operation.

The custom compiler uses unified programming method to compile the target code of the main processor and the intelligent computing coprocessor. The compiled instructions are divided into ordinary instructions and dedicated intelligent computing instructions. The overall calculation is performed in the form of an instruction pipeline. The main processor first uses the first-level decoding unit to decode the fetched instructions. The main processor then uses the second-level decoding unit to decode the fetched instructions and use the main processor for processing when it is an ordinary instruction. It is put into the AI instruction queue of the intelligent computing coprocessor to wait for processing when it is an intelligent computing instruction. The decoding unit for intelligent calculation decodes instructions and sends the decoded information to each calculation unit to control and achieve specific intelligent calculation operations.

The intelligent computing coprocessor mainly includes an AI instruction queue, an AI decode, a variety of AI computing units, a global buffer, a set of data registers which include vector registers and a set of parameter registers.

Instruction queue is used to temporarily store special intelligent enhanced instructions. The decoding unit decodes the fetched instructions and uses the decoded information to control other components. The parameter register file stores the fixed parameters for invocation.

Global buffer is a global data buffer unit. The hierarchical storage structure of intelligent computing coprocessor includes three parts: memory outside the chip, global buffer, and the data register file on the chip. Global buffer is a global data buffer unit. As the first buffer area in the chip, the input, weight data obtained from memory access and the output data that will be stored to memory are temporarily stored, which are the intermediate buffer units of the out-of-chip memory access data. The data stored in the data register file are the data of the calculation site. The input data and weight data are distributed to the data vector registers for calculation. The intermediate process data are also stored in the specified data register file, and the output data are collected into the global buffer according to the organization form.

The intelligent computing coprocessor computing unit consists of the main computing unit, the activation computing unit, and the pooling computing unit.

Main_CU is the main computing unit of the intelligent coprocessor, which implements convolution, full connection and and BN calculation. Convolution and fully connected operations share the main computing unit. At the same time, by adjusting the BN operation to a single MAC, the BN computing reuses the main computing unit. Activate_CU realizes the activation function. In our work, the hardware is achieved to use the vector register to complete the calculation of Relu, Relu6, and Lrelu. Pool _ CU is the pooling calculation unit of intelligent coprocessor, which supports maximum pooling and average pooling. When there is pooling operation behind the convolution layer, the data are organized on chip and the results of the convolution output feature map are stored in the buffer, which is directly used for pooling calculation to reduce the transmission of data inside and outside the chip.

### 4.2. Microarchitecture of SIMD Computing Unit

**Figure 5:**
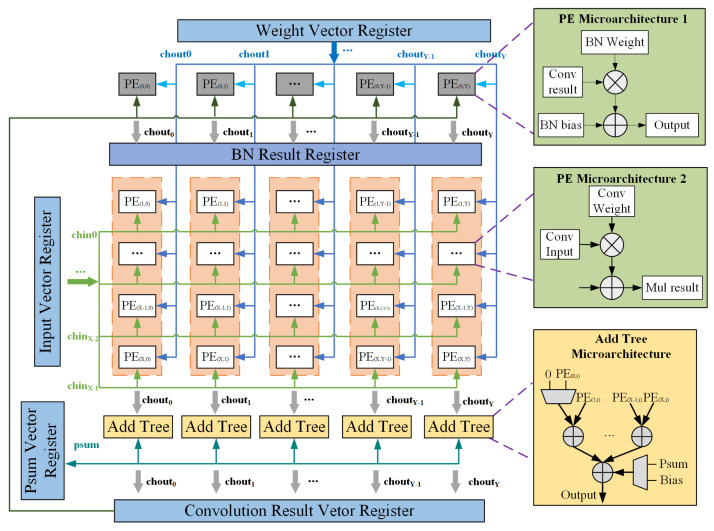
Microarchitecture of computing units for CONV calculation.

The main computing unit Main_CU adopts the SIMD architecture, which uses 8 × 8 PE arrays for parallel calculations, the PE array structure mapping is shown in Figure 5. For convolution and fully connected calculations, array rows are parallel for input channels, and columns are parallel for output channels. In other words, all of PEs are used in the form of microstructure 2. Different rows in each column remain the weight of different input channels corresponding to the same output channel. The weights of the same output channel corresponding to different input channels are used between different rows in each column. A column of PE calculated multiplication results are sent to the addition tree unit for accumulation. Each row of PEs maps the calculation of the same point in different output channels and reuses the neuron data of the same input channel.

The following takes a 3 × 3 convolution window and 8 input channels to calculate a pixel in an output feature map as an example to illustrate the calculation process.

1. Send the data corresponding to chin 0∼7 of the window_pixel (0,0) to 8 rows of PEs, and send the data corresponding to window_pixel (0,0) of chin 0∼7 which are corresponding to chout 0∼7 to PEs in rows 0∼7 and columns 0∼7, respectively. Calculate the partial sum of chout 0∼7, and then add it to the bias value in the bias register, that is, use bias as the initial part;
2. Send the data corresponding to chin 0∼7 of the window_pixel (0,1) to 8 rows of PEs, and send the data corresponding to window_pixel (0,0) of chin 0∼7 which are corresponding to chout 0∼7 to PEs in rows 0∼7 and columns 0∼7, respectively. Calculate the partial sum of chout 0∼7, and then add it to the previous partial sums;
3. In this way, until the sending of the data corresponding to chin 0∼7 of the window_pixel (2,2) to 8 rows of PEs, and the sending the data corresponding to window_pixel (2,2) of chin 0∼7 which are corresponding to chout0∼7 to PEs in rows 0∼7 and columns 0∼7, respectively. The MAC calculation results of a convolution window and 8 input channels are obtained.

When the number of the input channel or output channel is more than that of computing units, the calculations are divided into multiple input blocks, and the block switching sequence can be configured through instructions. For example, after calculating the K lines of an input block to obtain the output part sum of one line of the output block, change the K lines of the next input block to calculate the K of the next input block rows and the partial sums that have been obtained by calculating the current input block, and so on, until a complete line of the output block is calculated, and then start calculating the next line output data of the output block.

When the input channel or output channel is less than the number of computing units, instructions can be used for scheduling to group the rows or columns of the PE array and perform calculations at multiple pixels to improve resource utilization.

For BN calculation, since there is no high requirement for the calculation performance at the edge but for the limitation on the area and power consumption, it can be considered not to set BN calculation unit, but to reuse PE unit. In other words, the first row of PEs is used in the form of microstructure 1 for BN calculation while the rest are still used for convolution calculation of seven input channels in the form of microstructure 2.

The parameters (weight, bias, bn_weight, bn_bias) required for convolution or fully connected calculations and BN calculations are read in the parameter buffer. The input data required for the convolution calculation are the neuron value read from the buffer, and the input data required for the BN calculation are the convolution calculation results obtained from the previous clock or the convolution calculation results read from the buffer.

MAC, the result of the convolution calculation, is either used as a partial sum and directly involved in the next operation, or written out to the buffer and read when needed for subsequent calculations. The BN calculation result is directly sent to the subsequent calculation unit for activation and pooling calculation.

## 5. VLIW Instruction Set Architecture

The standard instruction set defined by RISC-V architecture only uses a small number of instruction coding spaces, and more instruction coding spaces are reserved for users as extended instructions. Therefore, the special VLIW instruction in this paper is based on RISC-V Custom instruction to extend the coprocessor.

### 5.1. VLIW Instruction Format

All instructions contained in the dedicated intelligent extension instruction subset are shown in Table 1. The instruction is fixed-length, but its function is variable-length. The dedicated intelligent extension instruction subset includes three categories of instructions, namely, intelligent computing instruction, data transmission instruction, and data movement instruction.

The data transmission instructions are designed for controlling the bidirectional dataflow between memory and global buffer. MEMRE represents reading the feature map data from memory. MEMWR represents writing the feature map data to memory. MEMWE represents reading the weight data from memory. Since the weight data are covered, there is no need to write back.

The data transfer instructions are designed for controlling the bidirectional dataflow between the data register file and global buffer. MOVIN represents moving the data from global buffer to the vector register file. MOVOUT represents moving the data from the vector register file to global buffer.

Intelligent computing instructions are designed for specifying computing types and controlling the dataflow in calculation site. CONV represents convolution computation. CONVBN represents convolution computation and batch normalization. POOL represents pooling computation.

**Figure 6:**
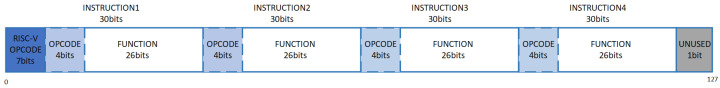
VLIW instruction format.

The VLIW instruction format of the dedicated intelligent instruction is shown in Figure 6, and the instruction length is 128 bits. According to the instruction encoding rule, the sixth to zero bit interval of the instruction is the opcode encoding segment specified by RISC-V. We set [6:0] to 0001011 to indicate the use of custom-0 command group for expansion. Each VLIW instruction contains four instruction slots, which can be filled with different dedicated intelligent extension instructions. Different extension instructions in instruction subset are distinguished by four bit opcode.

In addition to the complete instruction set in RISC-V that can be used for extension, each instruction also reserves a customizable coding space. In addition to the index used for register operands, there are many bits of coding space left. RISC-V custom instructions are used to configure the storage start address, the operation type included in the DNN layer, the input and output feature map size, the size of the convolution kernel, the number of input and output channels and the convolution stride, convolution pad, pooling stride, pooling pad, activation type, and other parameters. The configuration information is directly stored in the parameter registers of the intelligent coprocessor for call.

**Figure 7:**
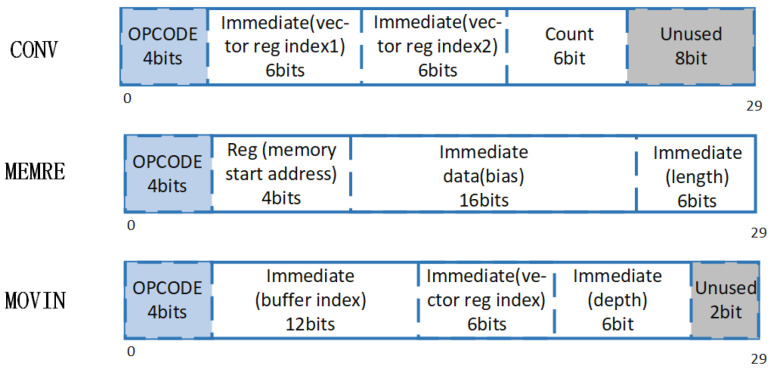
Some instructions in the dedicated intelligent extension instruction subset.

The format of some instructions in the dedicated intelligent extension instruction subset is shown in Figure 7. The design of the instruction is mainly focused on the dataflow maintenance at the coprocessor side. Adaptive structural mapping is carried out for different dataflows, and the overall control scheduling of the calculation process is realized. The dataflow in the whole acceleration process is mainly deployed around the out-of-chip memory space and the hierarchical storage structure in the chip.

The data transmission instructions describe the bidirectional dataflow between memory and global buffer, and realize variable block transmission by registers and bias addressing. The transmission contents include input, output, weight, and psum. Taking the data block as the basic unit of data stream organization, the data are divided into blocks and scheduled according to the data block. Through the address indexes of the off-chip data blocks, the data block switching sequence (loop execution sequence) is flexibly scheduled, so as to realize the adaptive control scheduling of the calculation.

The data transfer instruction focuses on the on-chip part of the hierarchical storage structure, describing how the dataflow distributes to the data register file from global buffer.

Intelligent computing instructions specify computing types, focusing on describing all structural parameters of the dataflow between the data vector registers and the computing components to obtain from the parameter registers with a fixed path and adjust the calculation state. The count operands in the instruction are set to reduce the overhead of complex control circuits. Only a few simple counting circuits are retained to implement small loop bodies.

### 5.2. Neural Network Application Deployment

VISA is a program-oriented architecture, which realizes the configuration and mapping of calculation through programs. We use the method of program to achieve deployment. The deployment means is VLIW scheduling, and the processor realizes NN through the operation of software.

#### 5.2.1. Scheduling of VLIW Instructions

The system pipeline has made some adjustments to suit this design. Inevitably, some 128-bits intelligent extended instructions will become unaligned due to the length of standard RISC-V instructions is 32-bits. In order to save an additional fetch cycle, we learn from [26] to deal with the problem of unaligned accesses. By using an additional register that retains the last instruction, 128-bit intelligent extension instructions can be easily fetched in one cycle even in unaligned cases.

Many parameters need to be modified to deploy different DNNs on VISA. During DNNs loading, computing, and storing data, the corresponding instructions are set according to network and structure parameters, as shown in Table 2.

By filling VLIW instruction slot flexibly with special intelligent extended instruction subset, the accelerated calculation of neural network can be easily realized. Take a 104 × 104 × 16-to-104 × 104 × 32 convolution layer as an example, and the instruction usage statistics are shown in Table 3. Since the establishment time of transmission protocol is uncertain during the execution of transmission instructions, the number of execution cycles is not a fixed value.

For a convolution computing instruction control process, in the initial stage, only the data transmission instructions and data transfer instructions are needed. When the on-chip data are sufficient to support the operation, convolution calculation is started, and then the data are collected until the calculation is completed and the data are saved back to the off-chip memory.

**Figure 8:**
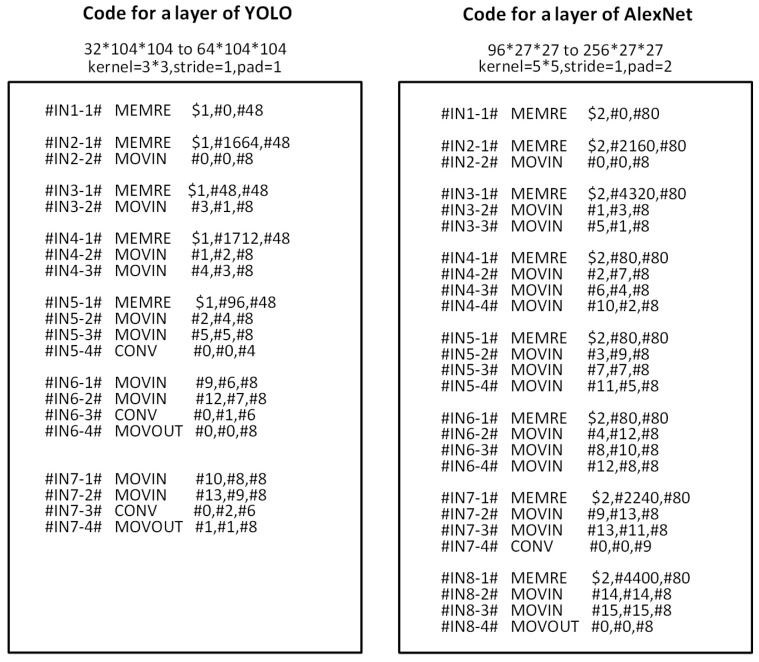
Examples of convolution calculation code.

The scheduling of the instructions maintains the dataflow. For example, Figure 8 shows the instruction control process of a layer in AlexNet and YOLO, respectively. In AlexNet, the input feature map size is 96 × 27 × 27, and the output feature map size is 256 × 27 × 27. In YOLO, the input feature map size is 32 × 104 × 104, and the output feature map size is 64 × 104 × 104. Therefore, flexible dataflow strategy can be realized to achieve performance improvement.

#### 5.2.2. Program Framework

**Figure 9:**
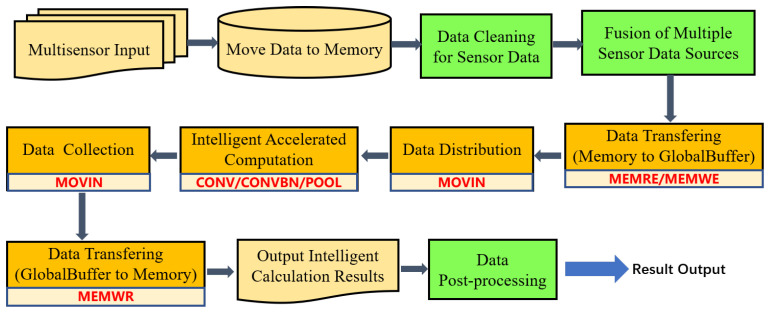
VISA program framework.

In the intelligent sensing system with multi-sensor, the running program can be divided into three main parts: data preprocessing, neural network acceleration calculation, data post-processing. The input data are first transferred through the bus from each sensor source to memory, shown in Figure 9. In particular, when mixing of various sensor data occurs, data cleaning, and data fusion are needed. Then the intelligent coprocessor completes data loading, data distribution, intelligent computing, data collection, data storage, and other steps. Finally, according to different task requirements, such as target recognition or image classification, the corresponding post-processing operation is carried out until the whole intelligent computing task is completed.

## 6. Experimental Evaluation

### 6.1. Design Synthesis and Resource Analysis

We implemented VISA in 45 nm GSCL technology with standard cell at 500 MHz using standard cell library and has been synthesized with Synopsys Design Compiler 18.06. Our design with RISC-V core and intelligent coprocessor implements the standard five-stage pipelined CPU based on RISC-V instruction set architecture, which supports standard RISC-V instructions includes RV32I∖M∖A∖F∖D. As is shown in Table 4, the detailed technical features of the VISA and the other hardware architectures are listed below.

### 6.2. Performance Evaluation

**Figure 10:**
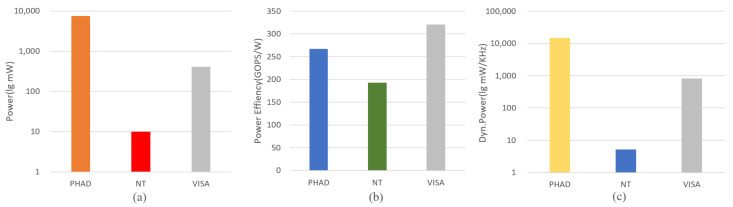
(**a**) Power comparison with intelligent processor. (**b**) Power efficiency comparison with intelligent processor. (**c**) Dynamic power with intelligent processor.

We take the proposed structure with several previous advanced edge-oriented architectures which are driven by instructions as a comparison. As shown in Figure 10, when compared with the instruction-driven intelligent architectures, in the processing of multiple different network structures, the performance and energy efficiency ratio of our proposed structure have been greatly improved compared with the previous structure, and the power consumption and delay are lower than the previous structure. Compared with PHAD, the power efficiency ratio is increased by 1.2×, while the total power consumption is also optimized. Compared with NT, although total power consumption is relatively higher, the power efficiency ratio is increased by 1.7×. Based on the limitation of realistic conditions, it is a reasonable trade-off between low power consumption and high computational performance.

In order to better study the performance improvement brought by the instruction-driven tightly coupled structure, we compared it with the intelligent accelerator LARENA [27] (baseline) of the same scale that we have implemented before. The two architectures have the same number of parallel computing units and support the same basic DNN operations. The difference is that the baseline uses hardware dynamic scheduling, but the structure proposed in our work is driven by instructions. In addition, BN is performed through a separate module in the baseline, but the structure proposed in our work combines and adjusts BN and convolution parameters, and reuses the main computing unit.

**Figure 11:**
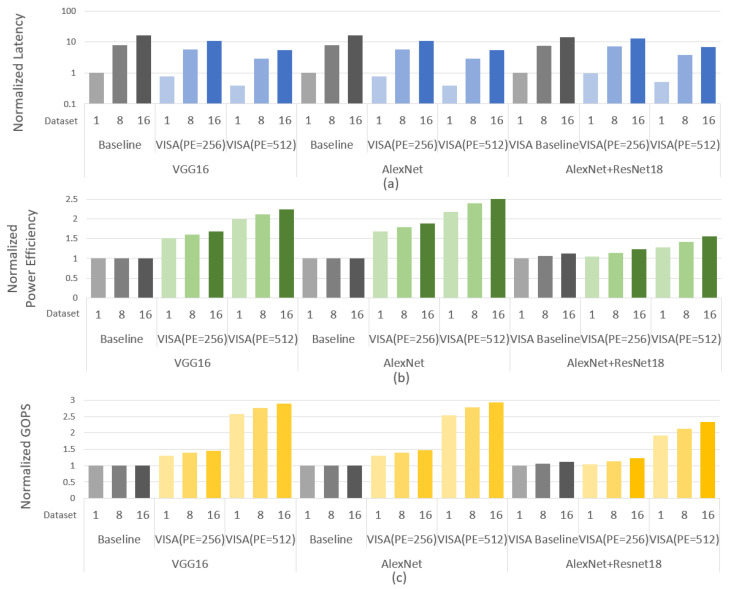
Performance comparison with baseline. (**a**) Normalized latency. (**b**) Normalized power efficiency. (**c**) Normalized throughput.

Figure 11 exhibits the latency, power efficiency and GOPS evaluation results for VGG16 and AlexNet when the PE number and dataset size vary. Under the same parallelism (256), the average and max speed-up of VISA are ×1.37 and ×1.45, respectively compared with the baseline. Meanwhile, the throughput and power efficiency increase by 37.45% and 68.44% on average. When the number of PE increases from 256 to 512, the average and max speed-up are ×1.88 and ×1.92, respectively. The throughput and the power efficiency ratio of VISA in the larger size effectively improve by 123.10% and 68.44%. Due to the bandwidth constrains for the memory-sensitive layers, the performance improvements are non-linear with the scale-up of PE array. With the enlargement of the dataset, the latency dramatically rises by 650%. Similarly, the throughput and power efficiency are improved by 6.7% and 6.8% on average. Because of the lacking of the scheduling mechanism for different datasets, the performance of the baseline remains more or less unchanged in Figure 11b,c. VISA adopts adaptive scheduling strategies for different datasets. The increase in the size of dataset makes the scheduling space significantly expand. Therefore, the resource utilization of VISA which uses scheduling strategies obviously increases, and the performance improves as well.

To better analyze the performance of multi-sensor input, we compare the performance of running AlexNet and ResNet one by one on VISA (VISA baseline) and computing two sensor data sources simultaneously. As is shown in Figure 11, compared with sequential calculation, the latency of computing multi-sensor data at the same time reduces 3.8% when dataset size is 1. When applying multi-sensor data sources on VISA, the computing and store resources are allocated based on different network scale. As resource allocation through fine-grained scheduling, the hardware utilization is improved for the multi neural network computation. In this scenario, the PEs which are not used due to data dependency are allocated to another network. In addition, with the increase in the dataset, the performance improves due to the scheduling space expands.

Under the same computing parallelism, the total computing latency is decreased because of the reduction in storage access delay. The reuse of the main computing for BN increases the calculation delay to a certain extent, but it can be seen from the Figure 11 that this increase have no discernible impact on total latency, which is still significantly reduced.

**Figure 12:**
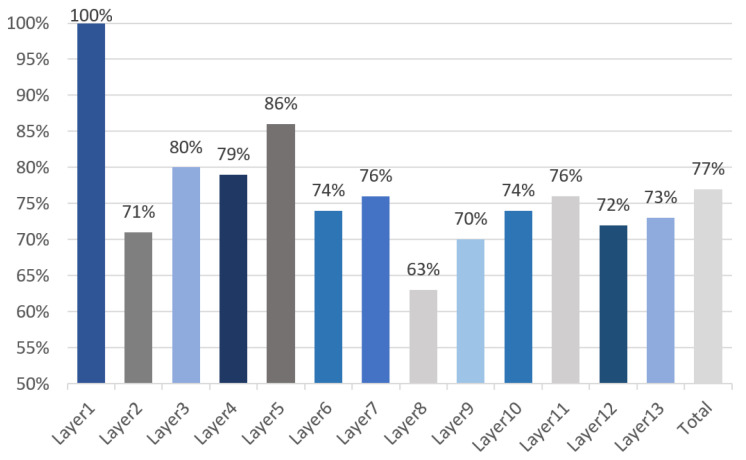
Memory access latency comparison with baseline.

As shown in Figure 12, the storage access acceleration ratios of each layer in the YOLO are analyzed. The data flow strategies of each layer are DI, DK, DK, DO, DK, DO, DK, DK, DK, DK, DO, DO, DI, respectively. Due to the support of various dataflow, VISA shows a 1.3× overall improvement compared with the baseline.

**Figure 13:**
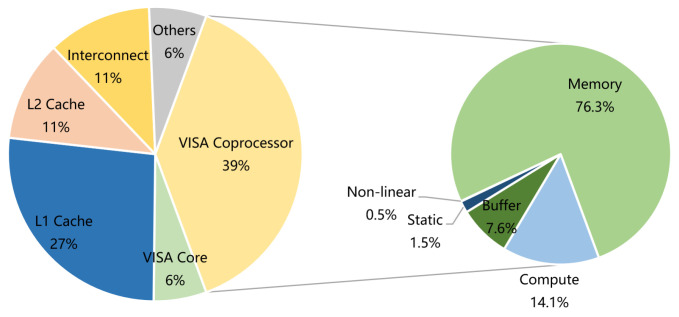
Power consumption of each part.

The power consumption of each part is shown in Figure 13, in which the total power consumption is 411 mW. On the whole, while the core of VISA contributes little more power (5%), the higher power consumption is mainly due to the frequent data accesses (38%), and the coprocessor operations (39%). The proportions of the total power consumption of VISA coprocessor are 76.3% (for off-chip memory), 14.1% (for calculation), 7.6% (for on-chip buffer), 1.5% (for static power) and 0.5% (for non-linear component), respectively. The off-chip memory access makes a significant contribution to the massive data transfer.

**Figure 14:**
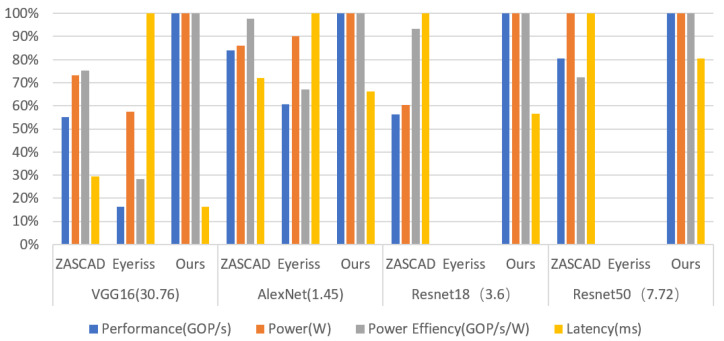
Performance comparison with accelerator.

We also compare the performance of VISA with some previous accelerators which are designed for very low power consumption conditions with hardware dynamic scheduling, as shown in Figure 14. VISA shows a little higher power consumption than the others because VISA runs at a higher frequency and there are much more PEs inside it. However, the overall power consumption is still within the allowable range (hundreds of milliwatts), satisfying low power condition. Notably, it can be seen from the figure that the performance and power efficiency ratio of the proposed instruction-driven tightly coupled architecture have been greatly improved compared with the previous hardware dynamic scheduling intelligent accelerator.

**Figure 15:**
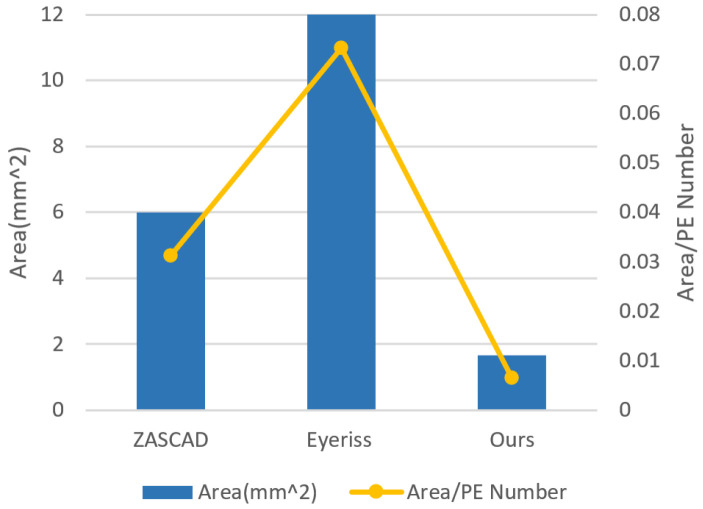
Area comparison with accelerators.

As is shown in Figure 15, VISA saves the area by 79% compared with Eyeriss and 91% compared with ZASCAD. By using the instruction extensions, the scale of control logic circuit is reduced, which plays an important role in the reduction in area.

## 7. Conclusions

In this paper, we propose a flexible configured pipeline integrated lightweight intelligent computing architecture VISA. In addition, we design a set of RISC-V dedicated intelligent enhanced VLIW instruction set for VISA. The goal of this work is to improve the computational performance as much as possible under the limit of low power consumption to obtain satisfactory energy efficiency ratio. Evaluation results show that the proposed VISA can provide power-efficiency improvement under the conditions of limited hardware resources.

## Figures and Tables

**Table 1:** The dedicated intelligent extension instruction subset.

Instruction Type	Example	Operand
Transmission instruction	MEMRE, MEMWR, MEMWE	Reg (memory start address)
		immediate (bias)
		immediate (length)
Movement instruction	MOVIN, MOVOUT	immediate (buffer index)
		immediate (vector reg index)
		immediate (depth)
Computing instruction	CONV, CONVBN, POOL	immediate (vector reg index1)
		immediate (vector reg index2)
		immediate (count)

**Table 2:** Instruction Setting.

Instruction	Network Parameters	Structure Parameters
MEMRE	input channel/input size/block/location	bandwidth/buffer size
MEMWR	output channel/output size/block/location	bandwidth/buffer size
MEMWE	input and output channel	bandwidth/buffer size
MOVIN	dataflow/stride/kernel size/padding	PE#/parallelism/reg size
MOVOUT	dataflow/block/location	PE#/parallelism/reg size
CONV	stride/kernel size/padding	PE#/reg size

**Table 3:** Cycle and Instruction Count.

Instruction Type	Cycles#	Instruction#
MEMRE	about 22,000	7280
MOVIN	35,776	35,776
CONV	192,200	21,632
MOVOUT	43,264	43,264
MEMWR	about 44,000	43,264
Sum	337,540	151,216
Average	67,508	30,243

**Table 4:** Resource Costs and Utilization.

Architecture	VISA	Eyeriss [2]	ZASCAD [27]	PHAD [16]	NT [26]
Technology (nm)	45	65	65	32	28
Voltage (V)	1	1	1	0.85/1.05	0.46
Frequency (MHZ)	500	200	200	500	40
Memory (KB)	132.5	181.5	36.9	1024	72 + 4
#PEs	256	168	192	4096	
Area (mm${}^{2}$)	1.66	12.3	6.0		0.068
Power (W)	0.411	0.257	0.301	7.5	0.001
